# Mitigation of endogenous oxidative stress and improving growth, hemato-biochemical parameters, and reproductive performance of Zaraibi goat bucks by dietary supplementation with *Chlorella vulgaris* or/and vitamin C

**DOI:** 10.1007/s11250-023-03657-6

**Published:** 2023-07-13

**Authors:** A. E. Abdel-Khalek, M. M. El-Maghraby, Zizy I. Elbialy, Rasha A. Al wakeel, E. A. Almadaly, M. Shukry, A. A. El-Badawy, H. K. Zaghloul, Doaa H. Assar

**Affiliations:** 1 Animal Production Department, Faculty of Agriculture, Mansoura University, Al-Mansoura, Egypt; 2 Animal Production Research Institute, Agricultural Research Center, Dokki, Giza, Egypt; 3 Department of Fish Processing and Biotechnology, Faculty of Aquatic and Fisheries Sciences, Kafrelsheikh University, El-Geish Street, Kafrelsheikh, 33516 Egypt; 4 Department of Physiology, Faculty of Veterinary Medicine, Kafrelsheikh University, El-Geish Street, Kafrelsheikh, 33516 Egypt; 5 Department of Theriogenology, Faculty of Veterinary Medicine, Kafrelsheikh University, El-Geish Street, Kafrelsheikh, 33516 Egypt; 6 High Institute for Agricultural Co-Operation, Shoubra, Egypt; 7 Department of Clinical Pathology, Faculty of Veterinary Medicine, Kafrelsheikh University, El-Geish Street, Kafrelsheikh, 33516 Egypt

**Keywords:** Goat, Lipid peroxidation, Semen, Microalgae, Vitamin C

## Abstract

This study was conducted to explore the effects of dietary inclusion of *Chlorella vulgaris* (CV) or/and vitamin C (VC) on growth, hemato-biochemical parameters, oxidative and antioxidant status, reproductive hormones, and semen quality variables, and scrotal-testicular dimensions of Zaraibi goat bucks. Twenty sexually mature bucks (41.49 ± 0.91 kg BW) were randomly divided into 4 groups (5 bucks/group). The control group was fed the control diet, while the other three groups received a diet supplemented with VC (2 g/animal /day), CV (5 g/animal/day), and CV plus VC (the same levels), respectively, for 8 weeks (treatment period), and then semen was collected for 8 weeks. Results showed that dietary supplementation with CV-VC combination significantly increased the final body weight, weight gain, packed cell volume, hemoglobin, red blood cells, white blood cells, and lymphocytes; elevated serum total protein, globulin, testosterone, estradiol, superoxide dismutase, glutathione peroxidase with a significant reduction in Malondialdehyde in serum and seminal plasma. Also, the CV-VC combination significantly improved the ejaculate volume, total sperm output, sperm concentration, and live sperm, and reduced reaction time and sperm abnormality of bucks. Either CV or VC given separately or in combination, at the chosen levels, had no detrimental effects on animal physiological responses with normal hepatic and renal functions. Therefore, the CV-VC combination could be safely utilized as a dietary supplement in buck’s diets to improve antioxidant defenses, scavenge free radicals, and potentiate buck’s reproductive activities under normal conditions.

## Introduction

The animal growth and development of the reproductive system can cause oxidative stress due to a reduced antioxidant defense system and increased lipid peroxidation (Sikiru et al. 2019). The oxidative stress can negatively alter animal nutrient absorption and feed utilization efficiency and suppress immune function, thus compromising animal growth performance (Sikiru et al. 2019) and male infertility outcomes in humans and animals (Bansal and Bilaspuri 2010). In this context, strong correlations have been found between increased lipid peroxidation in bull ejaculate and different sperm parameters, including sperm dysfunctions, low sperm concentrations, poor sperm motility, abnormal sperm morphology, alterations in serum steroid hormone levels, disruptions of spermatogenesis, and loss of fertility in buffaloes (Lone et al. 2018). Mammalian spermatozoa are more vulnerable to lipid peroxidation, including a high amount of specific lipid composition of sperm cells such as polyunsaturated fatty acid, plasmalogen, and sphingomyelin (Vernet et al. 2004).

Improving goat productivity is a significant achievement, especially for the poor in rural areas in developing countries (Hassan et al. 2013). As antioxidants have beneficial effects on the process of spermatogenesis in rats (Atta et al. 2017), dietary antioxidant supplementation is a suggested way of mitigating endogenous oxidative stress directly via enhancing antioxidant enzyme activities or indirectly through quenching of free radicals (Wu et al. 2005). Microalgae supplementation for different species has been found to enhance total antioxidant capacity, stimulate the immune response, promote growth, and improve the fatty acid profile and protein source in dairy goat ration (Tsiplakou et al. 2017a) and broilers (Abdelnour et al. 2019).

*Chlorella vulgaris* (CV) is a green microalga cultivated with high productivity and can be used as a functional food or supernatural supplement for humans and animals for beneficial health impacts (Panahi et al. 2016). CV is one of the essential natural feed supplements for humans and many animal species and has a highly nutritive unicellular freshwater microalga that is documented as a safe alga by the Food and Drug Administration (Bauer et al. 2017). CV is a rich source of numerous valuable substances such as S-nucleotide adenosyl peptide complex, polysaccharides, carotenoids, polyphenols, vitamins, and minerals (Sikiru et al. 2019).

Beyond amelioration of stress-associated growth, CV has been documented to alleviate organ toxicities induced by chemotherapeutic agents such as paracetamol in rats (Abd El Latif et al. 2021); similarly, *Spirulina platensis* against diclofenac sodium in broilers (Mokhbatly et al. 2018), and for cutaneous wound healing potential in a rat model (Elbialy et al. 2021). Chlorella species exhibit antimicrobial, anti-inflammatory, immune-modulatory, and analgesic activities, and even an anticancer agent with anti-oxidative, anti-hypertensive, and hypolipidemic and hypoglycemic effects in animal and human studies (Abd El-Hack et al. 2019; Barkia et al. 2019).

Vitamin C (VC) is an essential water-soluble antioxidant that inhibits the oxidation of protein, DNA, and nitric oxide (Frei et al. 1989). VC is a cofactor associated with some oxygenases involved in synthesizing several substances like collagen, catecholamines, carnitine, and the metabolism of xenobiotics, tyrosine, and cholesterol (Combs 2008). It is involved in vitamin E recycling; α-tocopherol scavenges proxy radicals and reacts with both of them to form an α-tocopheroxyl radical, followed by recycling back of this radical to α-tocopherol by VC (May et al. 1998). Interestingly, VC can minimize the various metal’s cytotoxicity, toxic mutagens, and xenobiotics in testes of treated animals by its efficient antioxidant and reactive oxygen species (ROS) scavenging properties (Korany et al. 2019).

In ruminants, the dietary requirements of VC for ruminants have not been confirmed because ruminants can synthesize VC in the liver. The adequate requirements of VC have been investigated by the determination of plasma VC levels, but wide variations in VC values were reported (Akinmoladun 2022). Apart from different routes of VC administration, direct dietary addition of VC may be effective (Kim et al. 2012). Even though plasma ascorbic acid concentration is higher via treatment with coated VC than powdered VC in drinking water or in the diet (Hidiroglou 1999), a similarity in the effectiveness of oral or injection of VC administration was reported in comparing the impact of both VC treatment routes on the stress of goat transportation (Biobaku et al. 2018). The potential of VC in the management of stressful stimuli in ruminants was reported by Akinmoladun et al. (2020a, b). Some studies have reported a reduction in the level of blood VC in stressed animals (Ali 2000; Ranjan et al. 2005) which may be a result of decreasing VC endogenous synthesis with increasing the need for VC. Moreover, Macleod et al. (1999) stated that high milk-producing dairy cows may synthesize less VC.

The effective antioxidant action of CV in comparison either with VC alone or their combination on the goat buck’s physiological responses and reproductive performance was not considered enough. Therefore, this study was designed to explore the impacts of dietary CV alga or/and VC supplementation on the physiological responses of Zaraibi goat bucks through estimating hemato-biochemical markers, oxidative stress and antioxidant biomarkers in serum and seminal plasma, hormonal concentrations, and buck’s reproductive performance.

## Materials and methods

This study was carried out at Sakha Experimental Station (Kafrelsheikh Governorate), Animal Production Research Institute, Agricultural Research Center, Egypt. *Chlorella vulgaris* microalga in the form of dried green powder was purchased from the Institute of National Research Center, Cairo, Egypt. Vitamin C was obtained from El-Nasr Company for Chemicals, Abozabaal, Egypt.

### Amino acid profile of *Chlorella vulgaris*

Sykam Amino Acid Analyzer (Sykam GmbH, Germany) equipped with Solvent Delivery System S 2100 (Quaternary pump with flow range 0.01 to 10.00 ml/min and maximum pressure up to 400 bar), Autosampler S 5200, Amino Acid Reaction Module S4300 (with built-in dual filter photometer between 440 and 570 nm with constant signal output and signal summary option) and Refrigerated Reagent Organizer S 4130.

The stock solution contains 17 amino acids (aspartic, threonine, serine, glutamic, proline, glycine, alanine, cystine, valine, methionine, isoleucine, leucine, tyrosine, phenylalanine, histidine, lysine, arginine). All amino acid concentrations were 2.5 μMol/ml, except cystine (1.25 μMol/ml). The stock solution (60 μl) was diluted in a 1.5-ml vial with sample dilution buffer and then filtered using a 0.22-μm syringe filter and then inject 100 μl.

One gram of each sample was well-mixed with 5 mL hexane, and then the mixture was allowed to macerate for 24 h. After that, the mixture was filtered on Whatman filter paper no. 1, and the residue was transferred into a test tube where it was incubated for 24 h with 10 mL 6N HCl in an oven at 110 °C. After the incubation, the sample was filtered on Whatman filter paper no. 1, evaporated on a rotary evaporator, and then entirely dissolved in 20 ml dilution buffer. The first dilution was prepared from this solution by diluting 2 mL to 100 mL dilution buffer, filtered using a 0.22-μm syringe filter, and 100 μl was injected. According to the manufacturer’s instructions, the sample was tested using BCA Protein Assay Kit (Novagen).

### Animals and feeding system

Twenty clinically normal and sexually mature Zaraibi goat bucks with average live body weight (LBW) of 41.25 ± 0.91 kg and 24 months of age were used in this experiment. All bucks were healthy and clinically free of external and internal parasites. The bucks were housed in a hygienic pen and fed on berseem (*Trifolium alexandrinum*), concentrates, and water provided ad libitum. Bucks’ management, handling during the whole experimental period, and blood collection were performed with an expert veterinarian’s aid.

The experimental bucks were fed individually according to NRC (2007). The concentrate feed mixture (CFM) consisted of 40% wheat bran, 35% ground yellow corn, 19% decorticated cottonseed meal, 3% cane molasses, 2% limestone, and 1% common salt. Bucks were weighed weekly, and the feed offered was adjusted based on body weight changes.

### Experimental design and diet

The experimental bucks (*n* = 20) were randomly allocated into 4 groups (5/group). The bucks in the 1st group (G1) were fed on the control diet (berseem and concentrates) without any additives. Bucks of G2 were fed the control diet supplemented with *Chlorella vulgaris* (CV) at a level of 5 g/kg concentrates, while those in G3 received the control diet supplemented with vitamin C (VC) in the form of ascorbic acid at a level of 2 g/kg concentrates**.** Bucks in G4 received a diet supplemented with the same levels of both CV and VC mixed with concentrates. The experimental period (16 weeks) included a treatment period of 8 weeks, followed by a semen collection period of 8 weeks.

### Biometry procedures

During the treatment period, the initial and final body weight of bucks was receded, and then the total weight gain was calculated. At the end of the semen collection period, the length (dorsal–ventral distance) and width (mid-lateral diameter) of the testicular mass (right and left side) were measured. The actual testicular volumes were determined by water displacement.

### Semen collection and evaluation

Semen ejaculates were collected from the bucks in the experimental (*n* = 5/group) twice/week for 8 weeks using an artificial vagina of goat bucks. Palpation of the external genitalia showed that they were typically regular. On the day of semen collection, reaction time, as the time elapsed from buck seeing doe until complete ejaculation, was estimated. Semen volume was measured by the graduated collection tube, and then the semen was evaluated for the percentages of initial sperm motility (Melrose and Laing 1970), sperm livability (Eosin and Nigrosin stain), and sperm morphological abnormality (different types of abnormality in head, neck, and tail of sperm cells). Sperm cell concentration (× 10^9^/ml) was estimated microscopically using a Neubauer hemocytometer, while the total sperm output (× 10^9^/ejaculate) was calculated by multiplying sperm cell concentration (× 10^9^/ml) by ejaculate volume (ml). Seminal plasma was obtained by centrifuging semen samples at 700 g for 20 min, and then seminal plasma samples were stored at − 20 °C until analysis.

### Blood sampling

At the end of the treatment period (8 weeks), two blood samples were collected from the jugular vein of each buck. The first blood sample was collected in a sterile tube with EDTA, as an anticoagulant, for hematologic parameters. The second blood sample was collected in a plain centrifuge tube, left to clot, then centrifuged at 3000 g for 15 min, and then serum samples were separated and stored as aliquots at − 20 °C for biochemical analyses.

### Hematologic and biochemical parameters

The hematologic parameters included red blood cells count (RCBs), packed cell volume (PCV), hemoglobin concentration (Hb), the white blood cells count (WBCs), and differential counts of WBCs by using automated blood cells counter with an Auto Hematology Analyzer (Sysmex F-800, Japan) according to Buttarello (2004).

Serum samples were analyzed for total proteins (TP) and albumin (Alb) concentrations according to Henry (1974), while globulin concentration (Glob) was computed by subtracting albumin from the total protein concentration. The concentrations of serum triglycerides (TG) and total cholesterol (TC) according to Richmond (1973) and high-density lipoprotein (HDL) cholesterol (Abell et al. 1952) were assayed. Low-density lipoprotein-cholesterol (LDL-C) and very low-density lipoprotein-cholesterol (VLDL-C) concentrations were calculated using the standard Friedwald equation (Friedwald et al. 1972). Concentrations of serum glucose (Trinder 1969), urea (Henry 1974), and creatinine (Fabiny and Einghausen 1971) were determined. Serum enzymatic activities of alanine aminotransferase (ALT) and aspartate aminotransferase (AST) were also determined in blood serum as described by Reitman and Frankel (1957).

All kits used for biochemical analysis were purchased from Biodiagnostics, Cairo, Egypt. All chemicals used in this study were of analytical grade.

### Lipid peroxidation, antioxidant biomarkers

Lipid peroxidation was evaluated by measuring malondialdehyde (MDA) content in blood serum and the seminal plasma (Ohkawa et al. 1979). Also, superoxide dismutase (SOD) was measured using the techniques outlined by Owens and Belcher (1965), while the activity of glutathione peroxidase (GPx) was evaluated according to Paglia and Valentine (1967) in blood serum and the seminal plasma. All kits were purchased from Biodiagnostics, Cairo, Egypt.

### Hormonal assay

Concentrations of testosterone and estradiol were determined in blood serum and the seminal plasma by the enzyme-linked immunosorbent assay (ELISA) using a commercial kit (DRG Diagnostics GmbH, Marburg, Germany) following the manufacturer’s instructions (DRG Diagnostics).

### Statistical analysis

Before analysis, data were tested for normality and homogeneity by Shapiro–Wilk’s and Levene’s tests, respectively. The GraphPad Prism computer software version 9.0 (GraphPad Software, San Diego, CA, USA) was used as multiple comparisons among means for hematological parameters, semen characteristics, lipid peroxidation markers, and antioxidant enzyme activity. One-way ANOVA followed by Tukey’s multiple comparison tests (post-hock test) were used to compare the mean of the experimental groups. An arcsine transformation was used before processing percentage data.

## Results

### Amino acid profile of *Chlorella vulgaris* powder

The individual values of each amino acid, as essential (EAA), non-essential (NEAA), and total in CV powder are presented in Table 1. Analysis of CV protein indicated that CV contained a variety of EAA and NEAA. Leucine (1.209%) as EAA as well as aspartic (1.603%), alanine (1.079%), and cysteine (1.083%) in NEAA is the most frequent in CV protein. The percentage of EAA was lower than that of NEAA (4.693 vs. 9.327%), representing an EAA to NEAA ratio of 0.503 and total amino acids of 14.019% in CV protein.

**Table 1:** Amino acid profile of *Chlorella vulgaris*

Essential amino acid (EAA)	%	Non-essential amino acid (NEAA)	%
Threonine	0.647	Aspartic	1.603
Valine	0.738	Serine	0.796
Methionine	0.272	Glutamic	2.086
Isoleucine	0.350	Proline	0.628
Leucine	1.209	Glycine	0.926
Phenylalanine	0.729	Alanine	1.079
Histidine	0.152	Cystine	1.083
Lysine	0.596	Tyrosine	0.440
-	-	Arginine	0.686
Total EAA	4.693	Total NEAA	9.327
Total profile	14.019	EAA/NEAA ratio	0.503

### Growth and scrotal-testicular dimensions

Feeding bucks on concentrates supplied with CV, VC, or their combination had significant (*P* < 0.05) affirmative influences on final body weight, weight gain, scrotal circumference, testicular length (right and left), and testicular volume as compared to the control group. A more positive effect on weight gain of bucks was found significantly (*P* < 0.05) for CV-VC combination than CV or VC treatment alone. However, right and left testicular width was not affected by treatments (Table 2).

**Table 2:** Live body weight and biometry of scrotum and testis (right and left) of bucks in the experimental groups

Item	Experimental group				
	Control	Vitamin C	Chlorella	Chl. + Vit. C	*P* value
Growth performance of bucks (kg)					
Initial body weight	41.7 ± 0.88	41.7 ± 1.45	41.3 ± 1.21	41.3 ± 0.88	0.792
Final body weight	49.3 ± 1.04^b^	51.3 ± 1.09^a^	51.2 ± 1.07^a^	53.2 ± 1.04^a^	0.041
Total weight gain (kg)	7.7 ± 0.16^c^	9.7 ± 0.26^b^	9.8 ± 0.26^b^	11.8 ± 0.31^a^	0.015
Scrotal and testicular biometry					
Scrotal circumference (cm)	29.3 ± 0.21^b^	31.0 ± 0.41^a^	31.2 ± 0.22^a^	32.3 ± 0.24^a^	0.045
Right testis length (cm)	11.2 ± 0.14^b^	11.6 ± 0.03^a^	12.2 ± 0.16^a^	12.8 ± 0.12^a^	0.031
Left testis length (cm)	10.8 ± 0.16^b^	11.4 ± 0.11^a^	11.6 ± 0.12^a^	11.9 ± 0.12^a^	0.010
Right testis width (cm)	6.6 ± 0.151	6.9 ± 0.230	6.9 ± 0.170	7.0 ± 0.151	0.382
Left testis width (cm)	6.6 ± 0.210	6.8 ± 0.181	6.8 ± 0.150	7.0 ± 0.110	0.194
Testicular volume (ml)	327.0 ± 0.31^b^	345.3 ± 0.26^a^	340.1 ± 0.28^a^	353.3 ± 0.41^a^	0.001

^a^^,^^b,c^Different superscripts among means on the same row indicate statistical significance differences at *P* < 0.05

### Hematological parameters

**Fig. 1:**
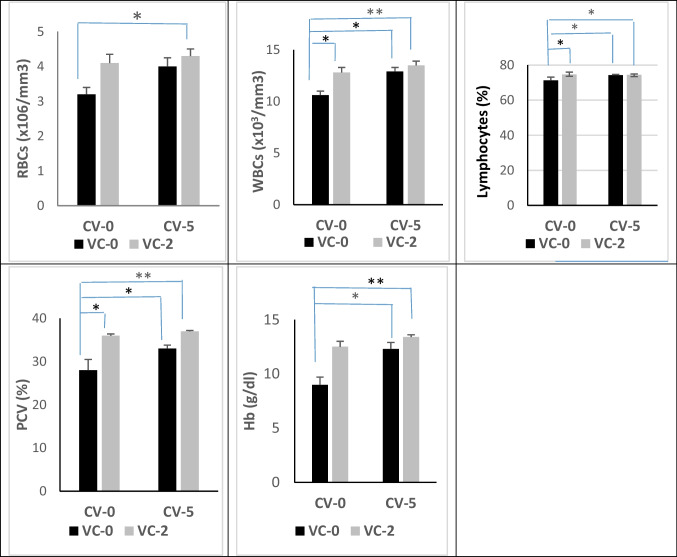
Hematological findings included red blood cells (RBCs), hemoglobin (Hb), packed cell volume (PCV), and white blood cells (WBCs) of bucks in the experimental groups. Data are expressed as mean ± SE (*n* = 5). *Significant differences at *P* < 0.05, **significantly differences at *P* < 0.01

In comparison with the control, as illustrated in Fig. 1, treatment with VC significantly increased PCV (*P* < 0.01) and count of WBCs and lymphocytes (*P* < 0.05). Treatment with CV significantly increased Hb, PCV, WBCs, and lymphocytes (*P* < 0.05). Treatment with CV-VC combination significantly increased all hematological parameters including Hb, PCV, WBCs (*P* < 0.01), RBCs, and lymphocytes (*P* < 0.05).

### Serum biochemical parameters

In this study, all treatments with VC, CV, and CV-VC combination significantly (*P* < 0.05) increased total proteins, globulins, and HDL-C concentrations, while a significantly (*P* < 0.05) reduced triglyceride (TG), total cholesterol (TC), VLDL-C, LDL-C, and glucose concentrations compared with the control group. The combination of CV and VC was significantly (*P* < 0.05) more effective than VC or CV alone on total proteins, globulin, triglycerides, total cholesterol, and LDL-C concentrations. On the other hand, all treatments showed non-significant effects on the serum activities of ALT and AST, creatinine, and urea concentrations (Table 3).

**Table 3:** Biochemical findings and enzyme activity of AST and ALT in blood serum of bucks in the experimental groups

Parameter	Experimental group				
	Control	Vitamin C	Chlorella	Chl. + Vit. C	*P* value
Total proteins (g/dl)	5.35 ± 0.058^c^	5.94 ± 0.053^b^	5.91 ± 0.056^b^	6.63 ± 0.130^a^	0.001
Albumin (g/dl)	2.96 ± 0.052	3.19 ± 0.078	3.04 ± 0.068	3.09 ± 0.116	0.281
Globulin (g/dl)	2.39 ± 0.098^c^	2.75 ± 0.125^b^	2.87 ± 0.078^b^	3.54 ± 0.172^a^	0.015
Triglycerides (mg/dl)	91.78 ± 2.377^a^	83.80 ± 3.412^b^	80.80 ± 3.99^b^	77.60 ± 3.172^c^	0.016
Total cholesterol (mg/dl)	105.4 ± 3.717^a^	94.87 ± 3.010^b^	90.23 ± 0.761^b^	86.40 ± 1.030^c^	0.014
HDL-C (mg/dl)	45.40 ± 1.448^b^	53.61 ± 0.094^a^	54.86 ± 1.099^a^	58.15 ± 1.960^a^	0.045
VLDL-C (mg/dl)	18.36 ± 0.420^a^	16.76 ± 0.630^b^	16.16 ± 0.781^b^	15.52 ± 0.641^b^	0.048
LDL-C (mg/dl)	41.64 ± 3.520^a^	24.501 ± 1.98^b^	19.21 ± 2.141^b^	12.73 ± 3.480^c^	0.003
Glucose (mg/dl)	59.89 ± 1.761^a^	49.80 ± 1.850^b^	45.20 ± 2.571^b^	41.200 ± 2.384^b^	0.045
Urea (mg/dl)	41.40 ± 0.812	39.20 ± 1.2	40.40 ± 1.764	38.60 ± 1.534	0.497
Creatinine (mg/dl)	0.978 ± 0.030	0.980 ± 0.068	0.990 ± 0.051	0.980 ± 0.065	0.209
AST (IU/L)	59.12 ± 0.488	61.70 ± 3.17	63.44 ± 2.129	62.81 ± 1.067	0.452
ALT (IU/L)	40.42 ± 1.771	47.77 ± 3.401	48.15 ± 2.880	49.41 ± 1.170	0.081

*HDL-C*, high-density lipoprotein cholesterol; *VLDL-C*, very low-density lipoprotein cholesterol; *LDL-C*, low-density lipoprotein cholesterol; *AST*, aspartate aminotransferase; *ALT*, alanine aminotransferase

^a^^,^^b,c^Different superscripts among means on the same row indicate statistical significance differences at *P* < 0.05

### Lipid peroxidation and antioxidant enzyme activity

#### In blood serum

**Fig. 2:**
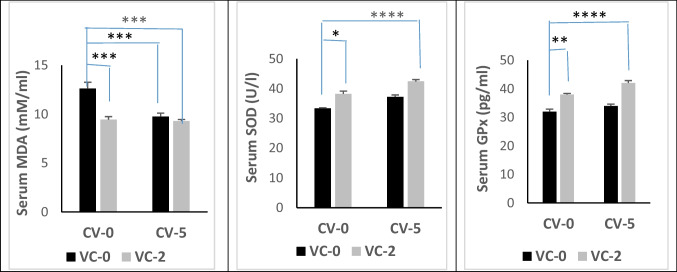
Level of malondialdehyde (MDA) and activity of superoxide dismutase (SOD) and glutathione peroxidase (GPx) in blood serum of bucks in the experimental groups. Data are expressed as mean ± SE (*n* = 5). *Significant differences at *P* < 0.05, **significant differences at *P* < 0.01, ***significant differences at *P* < 0.001, ****significant differences at *P* < 0.0001

Results illustrated in Fig. 2 indicated that dietary inoculation of CV or/and VC in a buck’s diet significantly (*P* < 0.001) reduced the level of MDA in the blood serum of bucks compared with the control diet. Treatment with VC significantly increased the activity of SOD (*P* < 0.05), and GPx (*P* < 0.01), but CV showed non-significant differences compared with the control.

However, the CV-VC combination succeeded in improving the serum antioxidant defense system via a significant enhancement (*P* < 0.001) in SOD and GPx activities, which was significantly higher than the effect either of VC or CV alone. These results revealed the superiority of VC alone or in combination with CV in comparison to CV in improving lipid peroxidation in the serum of bucks.

#### In the seminal plasma

**Fig. 3:**
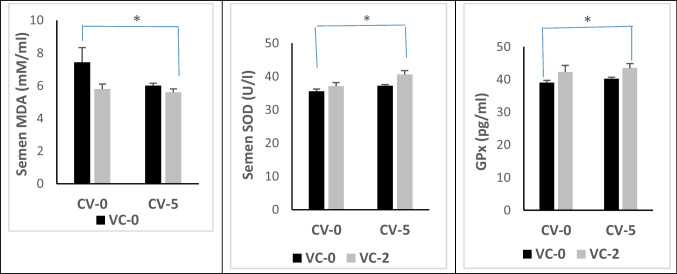
Level of malondialdehyde (MDA) and activity of superoxide dismutase (SOD) and glutathione peroxidase (GPx) in the seminal plasma of bucks in the experimental groups. Data are expressed as mean ± SE (*n* = 5). *Significant differences at *P* < 0.05

In this study, only the dietary inoculation of the CV-VC combination succeeded in improving serum antioxidant defense of the seminal plasma via a significant reduction in MDA level and significant enhancement in SOD and GPx (*P* < 0.05) compared with the control (Fig. 3).

### Hormonal profile

**Fig. 4:**
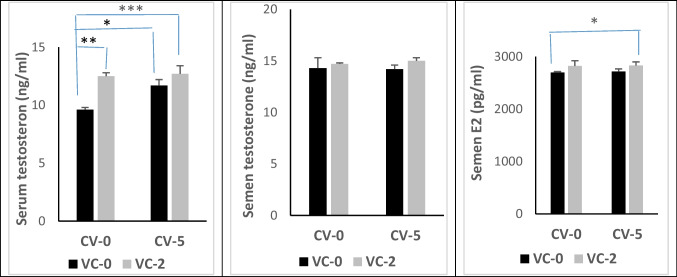
The concentration of testosterone in blood serum and semen, and estradiol (E2) in the semen of bucks in the experimental groups. Data are expressed as mean ± SE (*n* = 5). *Significant differences at *P* < 0.05, **significant differences at *P* < 0.01, ***significant differences at *P* < 0.001

The results of the current study also pointed out a significant elevation of testosterone levels in the serum of bucks fed diets incorporated with VC (*P* < 0.01), CV (*P* < 0.05), and their combination (*P* < 0.001) compared with the control group. However, the testosterone level in the seminal plasma of bucks was not affected by treatments. On the other hand, E2 concentration in the seminal plasma of bucks was increased significantly (*P* < 0.05) only by the CV-VC combination (Fig. 4).

### Reproductive performance

In the current work, we found a significant (*P* < 0.05) increase in semen variables including semen volume, initial sperm motility, progressive sperm motility, live sperm, sperm cell concentration, and total sperm output of bucks fed diets supplemented with all treatments. However, reaction time and abnormal sperm were significantly (*P* < 0.05) decreased by all treatments, but additional impacts were significantly (*P* < 0.05) observed for CV-VC combination on reducing the reaction time and abnormal sperm and also for VC on decreasing reaction time (Table 4).

**Table 4:** Sexual desire and semen characteristics of bucks in the experimental groups

Item	Experimental group				
	Control	Vitamin C	Chlorella	Chl. + Vit. C	*P* value
Reaction time (s)	57.3 ± 0.88^a^	37.0 ± 1.73^c^	42.6 ± 3.71^b^	36.0 ± 2.51^c^	0.004
Ejaculate volume (ml)	1.32 ± 0.361^b^	1.48 ± 0.252^a^	1.42 ± 0.330^a^	1.49 ± 0.240^a^	0.012
Initial sperm motility (%)	76.5 ± 0.25^b^	85.5 ± 0.21^a^	81.9 ± 0.29^a^	85.9 ± 0.31^a^	0.042
Progressive sperm motility (%)	67.9 ± 0.48^b^	79.4 ± 0.44^a^	85.6 ± 0.36^a^	87.6 ± 0.36^a^	0.035
Live sperm (%)	75.9 ± 0.26^b^	83.2 ± 0.15^a^	80.3 ± 0.55^a^	85.9 ± 0.13^a^	0.021
Abnormal sperm (%)	9.2 ± 0.01^a^	7.9 ± 0.06^b^	8.3 ± 0.19^b^	7.0 ± 0.40^c^	0.016
Sperm concentration (× 10^9^/ml)	2.78 ± 0.22^b^	3.06 ± 0.22^a^	2.95 ± 0.03^a^	3.21 ± 0.17^a^	0.001
TSO (× 10^9^/ejaculate)	3.72 ± 0.24^b^	4.36 ± 0.28^a^	3.99 ± 0.27^a^	4.38 ± 0.25^a^	0.001

^a^^,^^b,c^Different superscripts among means on the same row indicate statistical significance differences at *P* < 0.05. *TSO*, total sperm output

## Discussion

Generally, stress increases free radicals and pro-inflammatory molecule production inducing oxidative injury and inflammation and different metabolic disturbances (Srivastava and Kumar 2015). The current work declared that dietary supplementation of CV and/or VC exerted a significant (*P* < 0.05) increase in the final body weight, weight gain, scrotal circumference, and testicular length (right and left); the best results were recorded for the CV-VC combination. The growth promotion effect of microalgae was attributed to its containing high protein (25–50%), carbohydrates (5–35%), fat (5–20%) (Panahi et al. 2016), and mineral substances (5–10%) as well as carotene and vitamins (C and K and B) (Abdelnour et al. 2019). CV improves nutrients’ digestibility, utilization of absorbed nutrients, growth performance, immune-boosting, tissue rebuilding, antioxidant protection, and the intestinal microbial ecosystem (Abdelnour et al. 2019). Studying the amino acid composition of algae, like CV, might have great importance in determining their nutritional values. Our analysis indicated that CV has a significant content of both EAA and NEAA which could be an essential alternative source of protein in the animal diet (Mišurcova et al. 2014). Therefore, CV was suggested as a suitable feed additive for animals that could improve the amino acid profile of their diets. In ruminants, increasing VC level decreases oxidative stress markers (e.g., lipid hydroperoxide); hence, the requirement of exogenous VC addition is important (Kleczkowski et al. 2005). The daily dose of VC increased the final body weight of ram lambs under heat-stress condition (Abd-Allah and Zanouny 2014). Injected ascorbic acid (sodium ascorbate) before an 18-h (1675 km) transit drive increased the final body weight and average daily gain of steers (Deters and Hansen 2020). In our study, scrotal circumference and testicular measurements increased in parallel with the increasing live body weight of bucks indicating a positive relationship between testicular size and the body weight of animals (Madani et al. 2000).

In association with improving the growth performance of bucks, we found also a significant increase in hematological parameters including Hb, RBCs, PCV, WBCs, and lymphocytes by all supplements, especially CV-VC combination. Similarly, an elevation of RBCs and WBCs counts was reported in mice and dogs fed spirulina polysaccharides (Zhang et al. 2001). The enhanced RBCs, Hb, and WBC production may be due to the presence of phycocyanin and polysaccharide components in algae and may also be due to increased iron bioavailability via the reduction of ferric ions into ferrous ions by algae in mice (Wollenberg and Rummel 1987). Also, VC had a positive impact on animal hematological parameters. In this respect, dietary VC addition increased mean values of PCV, Hb, and lymphocytes, but neutrophils remained unchanged, in swampy buffaloes under heat stress (Konwar et al. 2017). VC actively protects lymphocytes and monocytes from oxidative damage (Carr and Maggini 2017). In goats, VC treatment (100 mg/kg body weight) eliminated the impaired effects of loading and transportation stress on the neutrophils, lymphocytes, and ratio of neutrophils to lymphocytes (Minka and Ayo 2011). VC treatment had an ability to protect neutrophils against oxidative stress and chemotactic responses of leukocytes in humans (Wolf 1993). Generally, VC improves humoral and cellular immunity leading to increasing resistance to infection and antioxidant status and reduces the deleterious effects of certain eicosanoids (Chambial et al. 2013). These findings indicating enhancement of the immune function of bucks by CV-VC combination in different ways.

In the present study, we detected enhanced total proteins and globulin concentrations in conjunction with improved unchanged albumin concentration by dietary CV-VC combination. This improvement may be attributed to the remarkable improvement the effect of CV as an anti-inflammatory and antioxidant with high levels of omega-3 PUFAs, numerous phenolic compounds, and β-carotene and vitamin B12 that altered immune responses through enhancing the production of both IgG and IgM in human (Barkia et al. 2019). Results of our study are in agreement with some authors, who found that plasma albumin in ram lambs were not affected by ascorbic acid treatment in summer season (Ghanem et al. 2008), while albumin of growing calves was increased by VC administration under heat stress (Kim et al. 2012).

In the current study, lipid profile was improved in terms of decreasing concentration of serum triglycerides, total cholesterol, VLDL-C, and LDL-C, and increasing HDL-C by all supplements, being the best for CV-VC combination compared with the control group. The hypocholesterolemia mechanism of CV-VC combination can be explained partially by the plausible decrease in acetyl–CoA enzyme fusion which is essential for fatty acid biosynthesis, or via inhibition of jejunal cholesterol absorption and ileal bile acid resorption (Nagaoka et al. 2005). The hypo-cholesterolemic actions of microalgae (Spirulina) involve reducing plasma and liver cholesterol via increasing the activities of lipoprotein lipase and hepatic triglyceride lipase (Karkos et al. 2008). In addition, α-glucan content in CV may act as an active immunostimulant, scavenging free radicals and reducing blood lipids (Iwamoto 2004). Also, VC can enhance the 7-α-hydroxylation of lipids and cholesterol nuclei, facilitating their breakage into bile acids to be easily eliminated from the body. Ascorbic acid has a hypocholesterolemic effect (Sahin et al. 2002; Yousef 2004), whereas VC increased plasma cholesterol but did not affect other blood biochemical of Rahmani ewes under heat stress (Hashem et al. 2016). On the other hand, plasma total cholesterol in ram lambs were not affected by ascorbic acid treatment in summer season (Ghanem et al. 2008).

The reduction in serum glucose level by VC or CV-VC combination was reported in stressed fishes supplemented with VC (Ortuno et al. 2003). In accordance with the obtained results, Kim and Kang (2015) reported a normal pattern of ALT and AST activities, creatinine, and uric acid following feeding fish with CV-supplied diets. On the other hand, VC treatment of ram lambs decreased the activity of AST and ALT (Abd-Allah and Zanouny 2014). These findings mean that dietary supplementation of CV and/or VC did not show any adverse effects on liver or kidney functions. Dietary CV or/and vitamin C offered protection against endogenous oxidative stress through scavenging free radicals, thus attenuating lipid peroxidation as evidenced by declined serum MDA levels besides enhanced activities of SOD and GPx. In the seminal plasma, the role of CV-VC combination was more effective on lipid peroxidation in blood serum than CV or alone. Microalgae contain natural antioxidant compounds such as phenols, flavonoids, carotenoids, and chlorophyll which enhance the antioxidant defense system and improve the antioxidant status in rabbits (Sikiru et al. 2019). Several factors can contribute to the antioxidant activities of microalgae (Spirulina and Chlorella); these factors include β-carotene, lutein, astaxanthin, bioactive peptides, phycocyanin, phenolic compounds, and sulfated polysaccharides (Barkia et al. 2019). At the same time, lycopene and phycobiliproteins present in the microalgae can serve as antioxidant agents, which could neutralize the enormous free radicals and prevent oxidative damage (Bhalamurugan et al. 2018). Furthermore, CV could increase selenium levels in the blood, thus enhancing the antioxidant status of laying hens under heat stress condition (Moradi et al. 2016). For other chlorella sp., Tsiplakou et al. (2017b) found that the daily intake of 11 g/goat from *Chlorella pyrenoidosa* has no positive impact in the antioxidant status of blood and milk (enzyme activity of SOD, CAT, and GPx as well as MDA level) of goat does. Concerning the effect of VC on antioxidant status, Nwunuji et al. (2014) found that VC (100 mg/kg, i.m) reduced the level of MDA in goat transported for 3.5 h. Thus, VC protects the body defense system and stabilizes animals’ health status by scavenging the excessive production of free radicals generated during stress. Vitamin C protective mechanisms may be through enhancing immune function via its role as an antioxidant that can easily donate electrons to free radicals and minimize the production of mutagenic metabolites because of its electrophilic nature, besides stimulating the production of interferon protein that protects cells from virus attack (Nitra and Silvia 2017). According to Belge et al. (2003), VC modulates the decrease in MDA concentration by removing the singlet oxygen, hydroperoxyl, superoxide, lipid peroxyl, and lipid-free radicals in animals subjected to stress. Besides the observed improvement in the seminal antioxidant status by reducing the level of MDA in bucks fed diet with CV-VC combination in our study, we declared also an impact on buck’s reproductive performance. The sexual desire of bucks was increased by reducing the reaction time and increasing testosterone levels in serum and seminal plasma. Semen quality was improved by increasing the ejaculate volume, sperm motility (initial and progressive), sperm livability, sperm cell concentration, and total sperm output while decreasing sperm abnormality compared with the controls. Also, levels of testosterone and estradiol hormones in serum and seminal were increased by CV-VC combination. Testosterone is a crucial hormone for regulating spermatogenesis and sexual behavior in the male reproductive system and for the normal physiology of seminiferous tubules in rats (Sharpe et al. 1994), while estradiol is biosynthesized by the action of aromatase enzyme mainly in the Leydig cells. Estradiol functions to control apoptosis of male sperm cells in human (Pentikainen et al. 2000). Milk antioxidant status was improved after feeding goats diets containing CV (Tsiplakou et al. 2017a). Moreover, CV supplementation can improve the physiological functions of both the brain and hypothalamus, leading to enhanced reproduction (Queiroz et al. 2016). The antioxidant properties of CV can influence spermatogenesis and sperm motility (Bansal and Bilaspuri 2010). A positive correlation between the oxidant/antioxidant capacity of buffalo bull ejaculate and the concentration and motility of sperm cells was reported by Lone et al. (2018). Supplementing a diet with a source of PUFAs-like commercial algal products has been found to enhance sperm output and sperm motility in bulls (Khoshvaght et al. 2016) and boar (Murphy et al. 2017). Sperm cells are more liable to oxidative stress; accordingly, to face this obstacle, CV supplementation may be advantageous because it contains both PUFAs and antioxidant substances such as polyphenolic compounds, β‐carotenoids, chlorophyll, ascorbic acid, phenolic compounds, α‐tocopherol, flavonoids, astaxanthin, and trace elements (copper, zinc, and magnesium) that are crucial for the function of antioxidant metalloenzymes (Kumar et al. 2014; Freitas 2017). Reactive oxygen species (ROS) can initiate cell injuries in different types of cells starting from spermatogonia up to mature sperm cells, consequently adversely influencing spermatogenesis, sperm morphology, sperm DNA integrity, and motility. Thus, oxidative stress may be the primary cause of impaired semen picture (Bansal and Bilaspuri 2010), and dietary CV or/and VC could improve semen quality and serum testosterone in buffaloes (Lone et al. 2018). Furthermore, the enhancement in sperm output could be attributed to improved testicular length and increased spermatogenesis in mice (Roqueta‐Rivera et al. 2010). Accordingly, it may be ascertained that testosterone, in addition to its high antioxidant activity, is involved in the mechanism by which CV acts (Kumar et al. 2014), as supplementation of CV has significantly enhanced the antioxidant status of the body and milk in goats as well as it improved antioxidant milk status (Tsiplakou et al. 2017a). Also, VC affects reproductive functions and synthesis of steroid hormones and has a correlation with the corpus luteum (CL) and plasma progesterone levels in the corpus luteum (CL) and plasma progesterone levels (Serpek et al. 2001). In Rahmani ewes, ovulatory follicle number, lambing rate, lamb weight, and fecundity were improved by VC treatment under heat stress condition. These improvements in the in vivo reproductive efficiency with VC were due to increased embryo viability and quality by (a) increased P4 production at the early stage of pregnancy, (b) improving placental, uterine, and oviduct functions, (c) improved blastocyte development, and (d) prevention of fetal resorption (Hashem et al. 2016). Moreover, in vitro studies indicated improvement in expansion and nuclear maturation of Caprine oocyte (Khanday et al. 2019) by ascorbic acid.

## Conclusion

Dietary supplementation of *Chlorella vulgaris* or/and vitamin C enhanced the antioxidant properties and scavenged free radicals, thus attenuating lipid peroxidation, and therefore offered protection against oxidative stress. This may be recommended as a feed additive that could improve physiological responses and potentiate bucks’ reproductive performance under normal conditions. However, more research with varied *Chlorella vulgaris* and vitamin C feeding levels is needed to investigate the molecular elements of increasing productive and reproductive performance in big herds of animals.

## Data Availability

The authors confirm that the data supporting the findings of this study are available within the article and/or its supplementary materials.

## Declarations

All precautions were followed to diminish animal suffering during the experiment. This study was approved by the Administration Committee of Experimental Animals, Faculty of Veterinary Medicine, Kafrelsheikh University, Egypt (KFS2020-3).
